# Apathy, Executive Function, and Emotion Recognition Are the Main Drivers of Functional Impairment in Behavioral Variant of Frontotemporal Dementia

**DOI:** 10.3389/fneur.2021.734251

**Published:** 2022-01-13

**Authors:** Gada Musa Salech, Patricia Lillo, Karin van der Hiele, Carolina Méndez-Orellana, Agustín Ibáñez, Andrea Slachevsky

**Affiliations:** ^1^Neuropsychology and Clinical Neuroscience Laboratory (LANNEC), Institute of Biomedical Sciences (ICBM), Neurosciences Department, East Neuroscience Department, Faculty of Medicine, University of Chile, Santiago, Chile; ^2^Departamento de Neurología, Clínica Universidad de los Andes, Santiago, Chile; ^3^Geroscience Center for Brain Health and Metabolism (GERO), Santiago, Chile; ^4^Department of Neurology South, Faculty of Medicine, Universidad de Chile, Santiago, Chile; ^5^Unidad de Neurología, Hospital San José, Santiago, Chile; ^6^Health, Medical and Neuropsychology Unit, Leiden University, Leiden, Netherlands; ^7^Facultad de Fonoaudiología, Pontificia Universidad Católica de Chile, Santiago, Chile; ^8^Cognitive Neuroscience Center (CNC), National Scientific and Technical Research Council (CONICET), Universidad de San Andrés, Buenos Aires, Argentina; ^9^The Global Brain Health Institute, University of California, San Francisco, San Francisco, CA, United States; ^10^Institute of Neuroscience (TCIN), Trinity College Dublin, Dublin, Ireland; ^11^Latin American Brain Health Institute (BrainLat), Universidad Adolfo Ibáñez, Santiago, Chile; ^12^Memory and Neuropsychiatric Clinic (CMYN), Department of Neurology, Hospital del Salvador & University of Chile, Santiago, Chile; ^13^Servicio de Neurología, Departamento de Medicina, Clínica Alemana-Universidad del Desarrollo, Santiago, Chile

**Keywords:** frontotemporal dementia, functionality, activities of daily living, apathy, executive function, functional impairment, emotion recognition

## Abstract

**Background:** The cognitive and neuropsychiatric deficits present in patients with behavioral variant frontotemporal dementia (bvFTD) are associated with loss of functionality in the activities of daily living (ADLs). The main purpose of this study was to examine and explore the association between the cognitive and neuropsychiatric features that might prompt functional impairment of basic, instrumental, and advanced ADL domains in patients with bvFTD.

**Methods:** A retrospective cross-sectional study was conducted with 27 patients with bvFTD in its early stage (<2 years of evolution) and 32 healthy control subjects. A neuropsychological assessment was carried out wherein measures of cognitive function and neuropsychiatric symptoms were obtained. The informant-report Technology–Activities of Daily Living Questionnaire was used to assess the percentage of functional impairment in the different ADL domains. To identify the best determinants, three separate multiple regression analyses were performed, considering each functional impairment as the dependent variable and executive function, emotion recognition, disinhibition, and apathy as independent variables.

**Results:** For the basic ADLs, a model that explains 28.2% of the variability was found, in which the presence of apathy (β = 0.33, *p* = 0.02) and disinhibition (β = 0.29, *p* = 0.04) were significant factors. Concerning instrumental ADLs, the model produced accounted for 63.7% of the functional variability, with the presence of apathy (β = 0.71, *p* < 0.001), deficits in executive function (β = −0.36, *p* = 0.002), and lack of emotion recognition (β = 0.28, *p* = 0.017) as the main contributors. Finally, in terms of advanced ADLs, the model found explained 52.6% of the variance, wherein only the presence of apathy acted as a significant factor (β = 0.59, *p* < 0.001).

**Conclusions:** The results of this study show the prominent and transverse effect of apathy in the loss of functionality throughout all the ADL domains. Apart from that, this is the first study that shows that the factors associated with loss of functionality differ according to the functional domain in patients with bvFTD in its early stage. Finally, no other study has analyzed the impact of the lack of emotion recognition in the functionality of ADLs. These results could guide the planning of tailored interventions that might enhance everyday activities and the improvement of quality of life.

## Introduction

Frontotemporal dementia (FTD) is the second most frequent form of young-onset dementia (<65 years old onset) after Alzheimer's disease dementia (ADD) (1, 2). Furthermore, FTD accounts for 15.3% (6.7–29.6% range) of patients with young-onset dementia (3). The main clinical manifestation is the behavioral variant (bvFTD) (4), which is characterized by personality changes, disinhibition, apathy, lack of empathy, changes in eating habits, and stereotypical behaviors. In addition, patients with bvFTD present cognitive deficits, particularly in executive functions (5, 6).

The aforementioned cognitive and neuropsychiatric deficits underlie the functional changes observed throughout the course of the disease (7). These functional changes consist of a loss of independence and functionality in the activities of daily living (ADLs) in their different domains: basic ADLs (BADLs), instrumental ADLs (IADLs), and advanced ADLs (a-ADLs). BADLs are defined as the daily activities directly related to basic physiological and self-maintenance needs, including tasks like eating, using the toilet, or getting dressed (8), while IADLs include essential activities to maintain an independent life, such as managing finances, shopping, handling medications, or using public transport (9). Finally, a-ADLs are more complex and voluntary activities. They include participation in social, productive, and leisure activities, such as working, playing games, planning social events, going on holidays, and active participation in communities (10–12). However, it remains unknown *how* and *which ones* are the main cognitive and neuropsychiatric deficits that affect the functionality of these patients.

Most of the current studies have been conducted on patients with ADD, wherein a dissociation has been reported regarding the influence of neuropsychiatric symptoms and cognitive factors on functional impairment at different stages of dementia and on each ADL domain (13). In mild cognitive impairment and mild ADD, apathy, and depression are relevant predictors of functional impairment in both IADLs and a-ADLs (13–15), while for patients with mild to moderate ADD, the main predictors of functional impairment in BADLs and IADLs are cognitive abilities (13, 15).

In the case of bvFTD, both cognitive and behavioral features have been associated with functional loss. In terms of cognitive function, some studies have identified global cognition and executive function as relevant predictors of global functional impairment (7, 16, 17). With regard to behavioral factors, apathy has been identified as the most critical variable influencing functional performance (7, 16, 18). Other studies have reported that executive, visuospatial, and language functions in conjunction with less severe disinhibition, aggression, and night-time behavior symptoms are associated with functional impairment (18, 19). Nevertheless, no study has yet identified predictive factors of functional impairment for basic ADLs. Moreover, most of the studies have been focused on the analysis of instrumental ADLs in patients with bvFTD, setting aside advanced ADLs, which are the first to be affected.

Another important component of bvFTD is the impairment of social cognition, deficits of which are markedly present in patients with bvFTD (20, 21). Social cognition is defined as the ability to recognize how other people are feeling and make judgments based on their inferred thoughts (22), and it includes domains such as the theory of mind (the ability to infer the beliefs, intentions, and mental states of others), emotion recognition (identifying facial expressions of emotions), and attributional style/bias (the explanation of individuals to understand others' intentions concerning social events and interactions) (23, 24). Deficits in social cognition could be related to disabilities in ADLs, specifically in a-ADLs, since this domain is directly related to social skills and can interfere in the achievement of personal goals and resolution of social problems (25). There are scarce studies on the association of disorders of social cognition and functional impairment. Only one study has evaluated the influence of social cognition on the functionality of patients with bvFTD, which found that the performance of ADLs was more strongly associated with motivation than with emotion processing (26, 27).

The studies on functional factors associated with bvFTD have only addressed IADLs and/or global functional impairment (16, 18, 28, 29). To the best of our knowledge, no studies have addressed factors associated with impairment in a-ADLs. Thus, it remains unknown how the different levels of ADL complexity (basic, instrumental, and advanced) are influenced by different neuropsychiatric and cognitive factors. Moreover, despite the paramount relevance of impairment in social cognition in bvFTD, studies on the association between social cognition and ADLs in bvFTD are scarce.

The main purpose of this study was to explore the association between cognitive and neuropsychiatric features that might prompt functional impairment at the different ADL domains in patients with bvFTD and a group of healthy control subjects.

We hypothesize that the cognitive and neuropsychiatric factors that predict functional impairment in patients with bvFTD differ among BADLs, IADLs, and a-ADLs. Specifically, we anticipate that impairment in BADLs is predicted by a lower executive function performance, presence of apathy, and disinhibition, while impairment in both IADLs and a-ADLs are predicted by poor executive function, social cognition, and presence of apathy.

## Materials and Methods

### Design

The research design was exploratory, analytical, cross-sectional, retrospective, and non-experimental. Ethical approval for this study was obtained from the University of Chile ethical committee (FONDECYT project N° 1160940) and the Ethical and Scientific Committees of the East Metropolitan Health Service and the HCUCH (Fondecyt 1170010, 1130920 & FONDAP 15150012).

### Participants

The study sample consisted of 59 participants, divided into 27 early-stage patients with bvFTD (< 2 years of progress since its onset) and 32 healthy control subjects. The patients with bvFTD were referred from two public hospitals in Santiago, Chile: Complejo Asistencial Barros Luco and Hospital El Salvador. The clinical diagnosis was performed by two cognitive neurologists according to the current criteria for bvFTD (30). The healthy control subjects were recruited by dissemination through the University buildings and social media. They were matched by age, gender, and education level. The inclusion criteria for the controls considered Spanish-speaking participants older than 60 years of age. All the participants have a reliable proxy who had known them for at least 5 years. The proxy was someone who was able to provide information about ADLs performance, behavioral changes, and the patients' general medical history. For all the participants, the exclusion criteria included <4 years of education, underlying neurological or psychiatric illness that could affect cognition (except for patients with bvFTD), and sensory disturbances that could interfere with the neuropsychological assessment. All the participants and their caregivers provided informed consent in accordance with the Declaration of Helsinki.

### Measures

The operationalized variables of this study are described as follows:

1. Activities of daily living: They were measured using the Technology-Activities of Daily Living Questionnaire [T-ADLQ; (31)]. The T-ADLQ is an informant-based report composed of 33 items. It assesses the percentage of functional impairment for different ADLs, which are assembled into seven subscales (self-care activities, household care, employment and recreation, shopping and money, travel, communication, and technology). Each question is rated from 0 (no problem) to 3 (no longer capable of carrying out the activity). Furthermore, each item has an extra alternative for cases where the patient may never have done the activity before (ND—“Never did this activity”), stopped the activity before the onset of dementia (e.g., working), or for which the proxy did not have enough information to give an accurate response (DK –“Don't know”), which allows correcting the score to premorbid functioning, thus avoiding gender and cultural bias (32). The overall functional impairment and each subscale were scored based on the procedure developed by the authors of the scale (32) as follows:
  $$\begin{array}{l} \frac{\sum \text{Total Score}\left[\text{Except itemsND}/\text{DK}\right]}{3\times \text{numbers of items answered}}\times 100\\ \left[\text{Except items with ND}/\text{DK}\right] \end{array}$$

By doing so, the items rated as ND/DK were excluded, which ensures that the functional impairment score was based on the actual functioning of the patients in comparison to their premorbid performance. Higher percentage scores indicate a higher functional impairment and are graded as follows: “none to mild” (0% to 33%), “moderate” (34% to 66%), or “severe” (more than 66%) (32). As previously reported, the T-ADLQ is divided into three domains: BADLs, IADLs, and a-ADLs (8, 13):

1.1. BADLs percentage of functional impairment.
1.2. IADLs percentage of functional impairment.
1.3. a-ADLs percentage of functional impairment.

Detailed information about the instrument and the items used for each variable can be found in the Supplementary Material.

2. Cognitive functioning: The Chilean version of the Mini-Mental State Examination (MMSE) was used in order to assess the overall cognitive performance. This instrument has a maximum score of 30, where a higher score indicates better performance. The cutoff point for the Chilean version was 21/22 for the diagnosis of dementia (33).

Executive functions were evaluated with different cognitive tests. First, we used the Frontal Assessment Battery [FAB; (34)], which is a screening test composed of six items that assess different functions (conceptualization, mental flexibility, programming, sensitivity to interference, inhibitory control, and environmental autonomy). Each item is scored from 0 to 3 points, where a higher score represents better performance. This test shows suitable psychometric properties in the Chilean population (30). Second, we used the FAS and animal version of the Controlled Oral Word Association Test [COWAT; (35)] to assess cognitive flexibility. This test has good psychometric properties (36) and assesses both phonological and semantic fluency, in which the participant has 1 min to name as many words as possible that start with a certain letter (F, A, and S) or belong to a specific semantic category (e.g., animals). Therefore, higher scores denote better functioning. Finally, the Digit Span Backward Task (37) was applied to have an estimation of working memory. The task consists of repeating back a sequence of numbers in reverse order, wherein the sequence length increases progressively. The obtained score represents the maximum number of items properly retrieved. This task has also shown good psychometric properties in Chilean population (38).

To assess social cognition, the mini-Social cognition & Emotional Assessment [mini-SEA; (39)] was used. This test is the short version of the SEA test (40), which is composed of adaptations of two widely used tests: the Faux pas test (41) to assess the theory of mind, and the Picture of Facial Affect test (42) to assess emotion recognition. The Faux pas task includes 10 short stories, wherein the participant must read and identify if the main character has or has not committed a social faux pas. On the other hand, the emotion recognition task includes 35 faces, wherein the participant must recognize the correct emotion, among seven possible options (happiness, surprise, neutral, sadness, anger, disgust, and fear). Both tasks have a maximum composite score of 15 points and the sum of both composite scores provides the total score for the mini-SEA (39).

3. Neuropsychiatric symptoms: These symptoms were measured using the Chilean version of the Neuropsychiatric Inventory Questionnaire [NPI-Q; (43)], an informant rating questionnaire that assesses the presence and severity of 12 neuropsychiatric symptoms, such as delusions, hallucinations, aggression, depression, anxiety, euphoria, apathy, disinhibition, irritability, aberrant motor behaviors, night-time disturbances, and eating disturbances (44). The presence scoring is based on YES/NO answers, whereas the severity score is rated as follows: 1 (mild); 2 (moderate), and 3 (severe). For the regression analysis, only apathy and disinhibition were considered, which have been identified as clinically significant in bvFTD (45–47) and might have relevance to the ADLs impairment.

### Procedure

The participants were assessed between 2016 and 2019. The neuropsychological assessment was carried out by a specialized neuropsychologist in two sessions of 90 min each, during which different cognitive tests were applied. Furthermore, a reliable informant was asked to complete the T-ADLQ and the NPI-Q at home in order to examine the participant's functionality in the ADL and the presence and severity of neuropsychiatric symptoms. Prior to inclusion in the study, all patients and carers signed an informed consent form.

### Statistical Analyses

IBM Statistical Package for the Social Sciences (SPSS) Professional Statistics v.24 (48) was used for the data analysis. An exploratory analysis was carried out in order to identify the distribution of each variable, using the Kolmogorov-Smirnov test. Based on the said analysis, performances of healthy control subjects and patients with bvFTD were compared on both cognitive and functional measures. *T*-tests were used for group comparisons of the measures with normal distribution and Mann-Whitney *U* tests were used for the variables that were not normally distributed. Likewise, Pearson and Spearman correlation analyses were performed to study the relationship between the different ADL domains and the cognitive and neuropsychiatric variables. Due to the use of different executive tests, composite scores were formed with unit-weighted z scores by using the means and SDs of the control group. This allowed for the creation of two composite variables: i) “executive function” made up of the variables COWAT's FAS version, FAB total score, and the Digit Span Backward Task and ii) “global composite score” formed by the variables MMSE total score, mini-SEA total score, and the NPI-Q severity scale, which were used for further analysis. Finally, in order to predict the best determinants of ADL impairment in its different domains in patients with bvFTD, three separate standard multiple regression analyses were performed using the stepwise (backward) procedure. For the regression analyses, BADs, IADLs, and a-ADLs were considered as dependent variables and executive function, social cognition, presence of apathy, and disinhibition were considered as independent variables. *p*-values <0.05 were considered significant. Only three of the four possible predictors could be used per analysis, because of the small sample size of this research. To handle this, the predictors were selected in line with each hypothesis.

## Results

### Participants' Demographics and Clinical Characteristics

A total of 59 participants were included, with 32 healthy control subjects and 27 patients with bvFTD. The groups did not differ in terms of age [*t*_(57)_ = −1.7, *p* = 0.10] and education (*U* = 365, *z* = −1.03, *p* = 0.30). However, gender differences were identified between the groups [$\chi_{\left(1\right)}^{2}$ = 5.9, *p* = 0.02], with more presence of women in the healthy control group (*n* = 23; 71.9%) and more men among the patients with bvFTD (*n* = 17; 63%). Table 1 summarizes the main findings and group comparisons.

**Table 1:** Comparison of participant demographics and neuropsychological tests.

**Variables**	**Healthy Controls**		**bvFTD**		***t*-test**	**Mann-Whitney *U***
	**Median**	**IQR or SD**	**Median**	**IQR or SD**		
Age (years)	65.3	8.1	69.1	9.4	−1.7	–
Education (years)	13.0	4.0	12.0	8.0	–	305
Gender (M/F)	9/23		17/10		–	–
Basic ADLs (% impairment)	0.0	0.0	13.3	20.0	–	769^**^
Instrumental ADLs (% impairment)	0.9	9.7	45.2	41.7	–	811^**^
Advanced ADLs (% impairment)	13.8	27.7	61.1	28.6	–	796^**^
Global ADLs (Total T-ADLQ)	3.3	12.0	42.0	30.2	–	822^**^
MMSE	29.5	1.0	26.0	6.0	–	107^**^
Executive function						
Composite ^a^	0.0	2.7	−7.7	4.3	7.9^**^	
FAB	16.0	3.0	12.0	5.0	–	106^**^
Digit Backward Test	4.0	2.0	3.0	1.0	–	239^*^
COWAT—FAS fluency^a^	43.5	12.7	23.0	15.7	5.5^**^	–
COWAT—animal fluency^a^	21.3	5.5	9.6	4.8	8.6^**^	–
Social cognition						
mini-SEA emotion recognition composite score	12.0	1.7	10.1	3.4	–	130^**^
mini-SEA Faux pas identification composite score	14.3	2.3	10.9	4.9	–	106^**^
Total score mini-SEA	25.4	3.1	18.6	7.4	–	95^**^
NPI-Q severity score	1.5	2.5	12.0	6.0	–	199^**^
Global composite score^a^	−0.2	0.7	−1.0	1.9	2.2	–

*IQR, Interquartile range; bvFTD, behavioral variant of Frontotemporal Dementia; ADLs, Activities of Daily Living; MMSE, Mini-Mental State Examination; FAB, Frontal Assessment Battery*.

a *Variables normally distributed, therefore, results are presented in mean and SD*.

* *p < 0.01*.

** *p < 0.001*.

Regarding general cognitive performance, patients with bvFTD had lower scores on the MMSE than healthy control subjects (*U* = 107, *z* = −5.04, *p* < 0.001). With regard to executive functioning, patients with bvFTD had worse outcomes than healthy control subjects on the FAB (*U* = 106, *z* = −5.02, *p* < 0.001) and COWAT [categorical fluency: *t*_(57)_ = 8.6, *p* < 0.001; lexical fluency: *t*_(57)_ = 5.5, *p* < 0.001]. Similar results were observed for social cognition (mini-SEA total score: *U* = 95*, z* = 4.66, *p* < 0.001), in emotion recognition (*U*= 130, *z*= −4.23, *p* < 0.001), and Faux Pas identification (*U* = 106, *z* = −4.48, *p* < 0.001).

### Activities of Daily Living

The percentage of functional impairment in patients with bvFTD increased along with the complexity of ADLs, meaning that mild impairment was observed in BADLs (*Median* = 13.3; *IQR* = 20.0), followed by moderate impairments in IADLs (*Median* = 45.2; *IQR* = 41.7) and a-ADLs (*Median* = 61.1; *IQR* = 28.6). A similar direction was detected in healthy control subjects (BADLs: *Median* = 0.0; *IQR* = 0.0; IADLs: *Median* = 0.9; *IQR* = 9.7; a-ADLs: *Median* = 13.8; *IQR* = 27.7).

**Figure 1:**
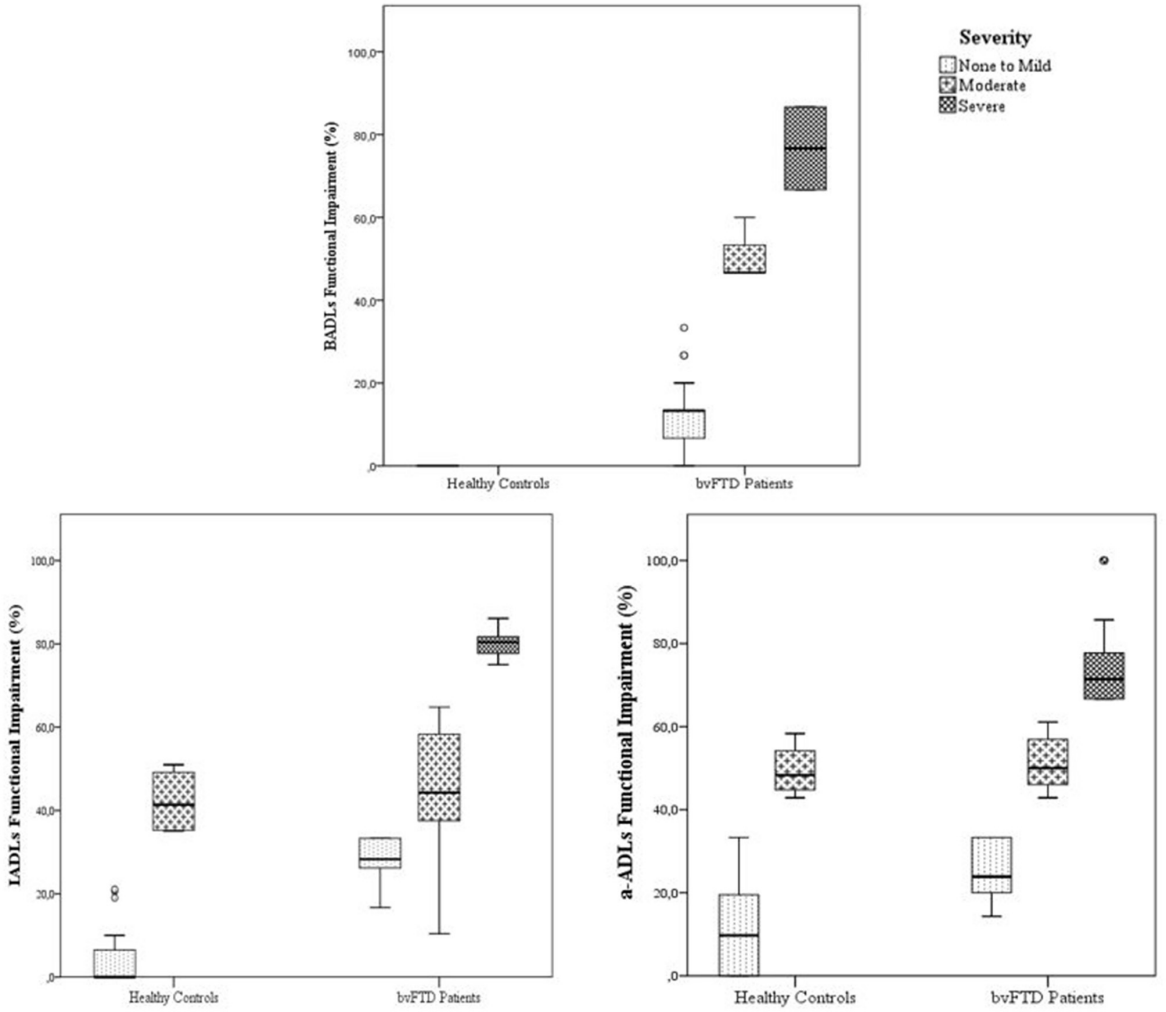
Boxplots with the distribution of participants percentage of functional impairment in BADLs, IADLs, and a-ADLs according to severity of impairment. The whiskers represent the range values of each group.

As expected, the bvFTD group showed higher levels of functional impairment in comparison with the healthy control subjects, and significant group differences were found in BADLs (*U* = 769, *z* = 5.61, *p* < 0.001), IADLs (*U* = 811, *z* = 5.83, *p* < 0.001), and a-ADLs (*U* = 796, *z* = 5.57, *p* < 0.001). In patients with bvFTD, a-ADLs and IADLs were the most affected, with 48.1% and 25.9% showing severe functional impairment and 29.6 and 51.9% showing moderate functional impairment, respectively. Figure 1 illustrates the distribution of the participants' functional impairment.

### Neuropsychiatric Symptoms

**Figure 2:**
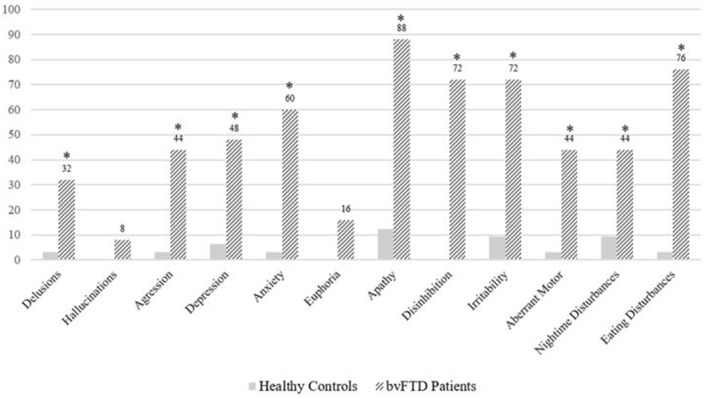
Prevalence of neuropsychiatric symptoms in healthy controls and bvFTD patients. *Significant difference between groups (*p* < 0.001).

Overall, there were significant differences between the control group and patients with bvFTD in terms of neuropsychiatric symptoms' severity score (*U* = 199, z = −5.06, *p* < 0.001). In terms of each symptom, apathy was the most frequent neuropsychiatric symptom observed in patients with bvFTD, reaching 88%. It was followed by eating disturbances (76%), disinhibition (72%), and irritability (72%). The frequency of all these symptoms, excluding hallucinations and euphoria, was significantly higher than the control group [6.7 > $\chi_{\left(1\right)}^{2}$ < 30.4, *p* < 0.001) (Figure 2).

### Correlations Between Cognitive and Neuropsychiatric Functioning and Functional Impairment at the Different ADL Domains

Overall, within all the participants, the three ADL domains were more strongly correlated with the neuropsychiatric variables (0.55 < *r*_*s*_ < 0.76, *p* < 0.01) than with the cognitive variables (−0.45 < *r*_*s*_ < −0.58, *p* < 0.01). Apathy was strongly correlated with the percentage of functional impairment across all ADL domains (BADLs: *r*_*s*_ = 0.68, *p* < 0.01; IADLs: *r*_*s*_ = 0.76, *p* < 0.01; a-ADLs: *r*_*s*_ = 0.72, *p* < 0.01), wherein disinhibition, emotion recognition, and executive function showed moderated correlations with the ADL domains (Table 2).

**Table 2:** Correlations (Spearman's Rho) for functional impairment of basic, instrumental, and advanced activities of daily living (ADL) with cognitive and neuropsychiatric features.

**Variable**	**N**	**Median**	**IQR^a^**	**1^b^**	**2**	**3**	**4**	**5**	**6**	**7**	**8**
**Functionality**											
1. Basic ADLs	59	0.00	13.30	–							
2. Instrumental ADLs	59	21.05	45.80	0.75^*^	–						
3. Advanced ADLs	59	33.33	47.20	0.59^*^	0.59^*^	–					
4. Global ADLs	59	19.79	38.54	0.77^*^	0.99^*^	0.89^*^	–				
**Cognitive performance**											
5. Executive Function Composite score^c^^,^^d^	59	−3.51	5.21	−0.46^*^	−0.58^*^	−0.53^*^	−0.59^*^	–			
6. Emotion Recognition composite score	56	11.57	2.60	−0.48^*^	−0.35^*^	−0.39^*^	−0.33^*^	0.53^*^	–		
**Neuropsychiatric symptoms**											
7. Apathy	57	0.00	1.00	0.68^*^	0.76^*^	0.72^*^	0.76^*^	−0.62^*^	−0.63^*^	–	
8. Disinhibition	57	0.00	1.00	0.59^*^	0.61^*^	0.55^*^	0.63^*^	−0.63^*^	−0.47^*^	0.59^*^	–

a *IQR, Interquartile Range*.

b *1, Basic ADLs; 2, Instrumental ADLs; 3, Advanced ADLs; 4, Global ADLs Cognitive performance; 5, Executive Function Composite score; 6, Emotion Recognition composite score Neuropsychiatric Symptoms; 7, Apathy; 8, Disinhibition*.

c *Results presented in mean and SD*.

d *Pearson r*.

*p < 0.01 (two-tailed)*.

* *p < 0.01*.

### Determinants of Functional Impairment in bvFTD

A preliminary analysis showed that mini-SEA total composed score and mini-SEA Faux Pas score did not impact functionality (advanced and instrumental ADLs) (49) (refer to the Supplementary Material for further details). Therefore, only the mini-SEA emotion recognition score was used as a potential predictor in the regression analyses.

For the regression analyses, BADs, IADLs, and a-ADLs were considered as dependent variables, and executive function (i.e., executive function composite score), social cognition (i.e., mini-SEA emotion recognition score), apathy, and disinhibition were considered as independent variables.

For a-ADLs, the best fit model explained 52.6% of the variance [*F*_(2,52)_ = 31.0, *p* < 0.001, adjusted *R*^2^ = 0.526]. This model included apathy and executive function. However, only apathy was a statistically significant factor (β = 0.59, *p* < 0.001), with a unique contribution of 33% of the variance explained.

With regard to IADLs, the best fit model explained 63.7% of the variance obtained [*F*_(3,51)_ = 32.6, *p* < 0.001, adjusted *R*^2^ = 0.637], and apathy, emotion recognition, and executive function were included (Table 3), wherein apathy (β = 0.71, *p* < 0.001), executive function (β = −0.36, *p* = 0.002), and emotion recognition (β = 0.28, *p* = 0.017) accounted for 66% of the variance explained (44% apathy, 17% executive function, and 4% emotion recognition).

**Table 3:** Standard multiple regression analyses with the percentage of functional impairment for basic activities of daily living (BADLs), instrumental activities of daily living (IADLs), and advanced activities of daily living (a-ADLs) scores as dependent variables.

**Predictor**	**B**	**SE**	**β**	***p*-value**	**sr^**2**^**
**Basic ADLs (BADLs)**					
Apathy	11.59	4.96	0.33	**0.020**	0.09
Disinhibition	11.35	5.42	0.29	**0.040**	0.08
**Instrumental ADLs (IADLs)**					
Executive function	−1.98	0.61	−0.36	**0.002**	0.17
Emotion recognition	3.47	1.41	0.28	**0.017**	0.04
Apathy	39.00	6.01	0.71	**<0.001**	0.45
**Advanced ADLs (a-ADLs)**					
Executive function	−1.12	0.64	−0.21	0.090	0.06
Apathy	32.59	6.38	0.59	**<0.001**	0.33

*B, Unstandardized regression coefficient; β, Standardized coefficient; sr, Semi-partial correlation squared*.

*Adjusted R^2^ for BADLs = 0.282, p < 0.001; adjusted R^2^ for IADLs = 0.637, p < 0.001; adjusted R^2^ for a-ADLs = 0.526, p < 0.001. Bold values indicates the significant results (p < 0.01 or p < 0.05)*.

Finally, for BADLs, the best fit model explained 28.2% of the variance obtained [*F*_(2,54)_ = 11.9, *p* < 0.001, adjusted *R*^2^ = 0.282] and included apathy (β = 0.33, *p* = 0.02) and disinhibition (β = 0.29, *p* = 0.04). Moreover, apathy and disinhibition uniquely contributed 9 and 8%, respectively, of the variance explained (Table 3).

## Discussion

The present study reveals that, in patients with bvFTD, the factors associated with functional impairment of the ADLs vary in their combinations and proportions across the different ADL domains. As expected, the performance of patients with bvFTD on all cognitive tasks and ADLs was significantly worse than the healthy control subjects, which is in line with several studies (28, 49, 50).

In terms of functionality, a-ADLs and IADLs were the most affected in patients with bvFTD. A similar pattern was observed in some healthy control subjects, which suggests that they could have other pathologies affecting their functionality. In the case of a-ADLs, almost half of the patients with bvFTD presented severe functional impairment (48.2%). Regarding IADLs, a similar proportion was observed (51.9%) in moderate functional impairment. These results are consistent with previous publications. For instance, Mioshi, Kipps (28) reported that 50% of patients with bvFTD have moderate impairments on IADLs. Interestingly, even if the patients of our study were in the mild and moderate stage of the disease, we observed that 67% of them reported mild impairment in BADLs and 18.5% reported moderate impairment in BADLs. This is also congruent with the findings of Mioshi, Kipps (28), who described marked impairment of both BADLs and IADLs in patients with bvFTD.

Concerning neuropsychiatric symptoms, 88% of patients with bvFTD presented apathy, 76% presented eating disturbances, and 72% presented disinhibition. These results are similar to those reported by Ranasinghe, Rankin (51). They described that in the mild stage of bvFTD, the most prevalent behavioral disturbances were apathy, followed by disinhibition and eating disturbances. Likewise, Johnson and Kumfor (52) found that 90% of patients with bvFTD presented apathy.

Regarding the factors associated with functional impairment, our regression model accounted for 28.2% of the BADL functional variability, wherein the presence of apathy and disinhibition plays a significant role. Contrary to expectations, poor performance on executive function does not contribute to the functional impairment of BADLs. Nonetheless, our results should be considered carefully since the patients analyzed were within the first stages of dementia, which usually presents scarce BADL impairment (53). Interestingly, Yassuda et al. have previously reported that neither cognition nor neuropsychiatric symptoms were associated with BADLs, measured with the Disability Assessment for Dementia (DAD), in bvFTD. Finally, further studies are needed to explore the factors associated with BADL impairment in patients with different severity stages of the disease.

Regarding IADLs, a model that explained 63.7% of the functional variability was produced, wherein the main contributors were apathy, executive function, and emotion recognition. These results are different from those reported by Yassuda, Lima da Silva (16). They obtained a model that explained 35.6% of the IADL variance, in which only global cognition acted as a significative predictor. One possible explanation is the fact that their sample was larger than ours; additionally, six out of seven of the predictors used in their model were behavioral. Moreover, they did not include specific measurements for executive functions and social cognition, which have been reported as the main domains impaired by bvFTD (20). Our findings are in line with the existing cognitive models since the tasks and activities that are supported by the executive functions play a central role in IADL performance (54).

Finally, the functional impairment of a-ADLs was best portrayed by a model that explained 52.6% of the variance, wherein only apathy was observed as a statistically significant predictor. This is the first study that explored the impact of cognition and neuropsychiatric symptoms in a-ADLs of patients with bvFTD. Thus, it was not possible to make direct comparisons with other research. Nevertheless, similar results were found in patients with ADD, wherein apathy was the strongest factor associated with both IADL and a-ADL impairment (13).

Overall, our study shows associations between the functional domains and other neuropsychiatric and cognitive factors, but it is not clear if these factors might affect a worse prognosis. One longitudinal study found that worse executive, visuospatial, and language functions in conjunction with more severe disinhibition, aggression, and night-time abnormal behavior symptoms also influenced a faster rate of functional impairment (19). In another longitudinal study, O'Connor, Clemson (18) examined 21 patients with bvFTD throughout 5 years, during which they observed that, while apathy symptoms increase, disinhibition with stereotypical behavior decreases during the disease progression. Even though they did not perform a prediction analysis, a longitudinal correlation was found between the detriment of these symptoms and a reduction of daily life functioning (18).

In terms of social cognition, emotion recognition was found to play a significant role in the functionality of instrumental ADLs. Our findings are in line with a study performed by Torralva, Gleichgerrcht (55), which concluded that in the early stages of bvFTD, emotion recognition deficits are significantly altered in comparison with Theory of Mind (ToM). This contrasts with the findings of Kipps, Mioshi (27), who did not find any relationship between emotion recognition and ADL performance in patients with bvFTD but was associated with the lack of motivation instead. Nevertheless, they used a different test to assess emotion recognition (the Emotion Hexagon). Another study concludes that social dysfunction in bvFTD appears to be multifactorial (25). Impairments in emotion processing may cause patients with bvFTD to be indifferent to social cues and thus, unable to respond to signals of social discontent. This deficit may prompt a lack of empathy or difficulty identifying situations that could embarrass them (27). In general, information is insufficient in order to conclude how social cognition deficiencies impact daily life functionality in bvFTD, and it should be explored in more detail. Nevertheless, this relationship has been examined in other pathologies, such as schizophrenia, bipolar disorder, traumatic brain injury, and Alzheimer's disease, where an independent contribution and significant correlations have been reported between social cognition, social behavior, and functional impairment (56–61).

Apathy was the main factor associated with functional impairment for all the ADL domains in patients with bvFTD. It showed a predictive power of 44% for the IADL functional variability and 33% for a-ADLs. These results are concordant with the findings of Yassuda, Lima da Silva (16), who showed that in patients with bvFTD, apathy, and global cognition act as predictors for global functional impairment. Moreover, similar outcomes were found in ADD, where apathy was the stronger predictor of functional impairment in both instrumental and a-ADLs (13). From a neurobiological perspective, it has been proposed that apathy involves three main domains: cognitive, affective, and behavioral (goal-directed), which have different underlying neural circuits (62). From a clinical perspective, the current diagnostic criteria for apathy include the following dimensions: **(i)** behavior and cognition, **(ii)** emotion, and **(iii)** social interaction (63). It is unclear how these three dimensions of apathy interact and influence functional impairment. Because of the strong effect that apathy has on functional impairment, future research is needed in order to improve the current comprehension of the underlying mechanisms of apathy on the functionality of ADLs. In order to do that, apathy should be assessed considering its multiple aspects, with the incorporation of different clinical instruments, such as behavioral tests, questionnaires, and even wearables. Currently, Zeghari, Robert (64) are working on a novel multidimensional protocol for apathy assessment in dementia, in order to achieve a better characterization of its different dimensions.

To date, this is the first study that has analyzed the functional impairment of a-ADLs in a sample of patients with bvFTD. This is clinically relevant since these activities are the first to be impaired once the disease starts its progression (53, 65). Nonetheless, there is a lack of studies that have addressed this dimension in patients with dementia. One of the possible reasons is the few instruments available to measure this construct. Currently, there are several tools available that have been designed and include a-ADLs as an exclusive type of ADL or join them together with other domains (8, 11, 12, 66, 67). More research is needed to increase the knowledge of this dimension and thus, incorporate the assessment of a-ADLs into clinical protocols, especially in those for the detection of early-onset dementias.

It is worth highlighting that our models only partially address functional impairment in ADLs, wherein apathy accounted for the prediction of <50% for the IADLs and a-ADLs' functional impairment, which implies that there are other factors that may influence the performance of each ADL domain. For instance, in a longitudinal study, Josephs, Whitwell (19) analyzed the contribution of cognitive, behavioral, genetic, and anatomical factors in the rate of functional decline in patients with bvFTD. As a result, they found that the atrophy pattern was the strongest predictor (*R*^2^ = 0.22) for a faster rate of functional impairment. Furthermore, there is a possibility that other manifestations of the disease, such as motor impairment, comorbidity, and sensorial deficit, could be interfering in the performance of ADLs.

The main limitation of this study is the small sample of patients with bvFTD, which implies that our results may not be generalizable, especially because bvFTD is a very heterogeneous disease. Further research with larger samples is needed to reach robust conclusions. In addition, with a larger sample size, other explanatory variables, such as perseverative behavior, eating disturbances, and irritability can be included that may provide models with a higher percentage of explained variance, and thus would contribute to the generalizability of the results. In the same way, there has been reported a high gender variability in patients with FTD (3, 68, 69), which also accounts as a limitation for the generalization of our results. Moreover, further studies should include other factors such as lack of insight and judgment problems, given that these are clinical characteristics of bvFTD (70–72) and may influence functional impairment of both IADLs and a-ADLs. Another limitation is related to the use of informant-based questionnaires for the assessment of functional impairment and neuropsychiatric symptoms, and the way it was conducted (at home), which could be susceptible to reporter bias. Nevertheless, despite these caveats, until today, they represent the best approach to evaluate functional impairment in dementia. This limitation could be overcome by carrying out clinical assessments such as semi-structured interviews with the patient and two close informants.

In summary, the present study found relevant clinical associations with functional impairment in the different types of ADLs. This study contributes to clarifying the association between some of the main cognitive and neuropsychiatric features present in patients with bvFTD and the different dimensions of ADLs. The main novelty of this study is the analysis of the functional determinants of a-ADLs in a sample of patients with bvFTD who are in the initial phase of dementia. The results provided have relevant clinical implications, which can guide the planning of early interventions and subsequent treatments. Moreover, early treatments could improve the quality of life, not only for the patients but also for their families and relatives.

## Data Availability Statement

The raw data supporting the conclusions of this article will be made available by the authors, without undue reservation.

## Ethics Statement

The studies involving human participants were reviewed and approved by University of Chile Ethical Committee (FONDECYT Project No. 1160940) and the Ethical and Scientific Committees of the East Metropolitan Health Service and the HCUCH (Fondecyt 1170010, 1130920 and FONDAP 15150012). The patients/participants provided their written informed consent to participate in this study.

## Author Contributions

GMS performed the neuropsychological assessments, built the database, and analyzed the data. PL and KvdH supervised the project. GMS wrote the manuscript with the support of KvdH, PL, CM-O, and AS. PL, AS, and AI referred subjects to the study. All authors provided critical feedback and helped to shape the research, analysis, and manuscript.

## Funding

PL, AS, and GMS were supported by ANID/FONDECYT REGULAR/1160940. GMS was sponsored by the scholarships program Becas Chile from ANID. PL, AS, and AI were supported by ANID / FONDAP/ 15150012. AI was partially supported by grants from Alzheimer's Association GBHI ALZ UK-20-639295, Takeda CW2680521, Sistema General de Regalías (BPIN2018000100059), Universidad del Valle (CI 5316) and CONICET. AS and AI are partially supported by ANID / Fondecyt/ (Nos. 1170010, 1171200, 1191726, 1210176, and 1210195), the Interamerican Development Bank (IDB), Multi-Partner Consortium To Expand Dementia Research In Latin America (ReDLat), National Institutes of Health, National Institutes of Aging (No. R01 AG057234), Alzheimer's Association (Nos. SG-20-725707 and GBHI ALZ UK-20-639295), Rainwater Charitable foundation - Tau Consortium, and Global Brain Health Institute. AS is partially supported by ANID/FONDEF/ (No. ID18I10113).

## Author Disclaimer

The contents of this publication are solely the responsibility of the authors and do not represent the official views of these institutions.

## Conflict of Interest

The authors declare that the research was conducted in the absence of any commercial or financial relationships that could be construed as a potential conflict of interest.

## Publisher's Note

All claims expressed in this article are solely those of the authors and do not necessarily represent those of their affiliated organizations, or those of the publisher, the editors and the reviewers. Any product that may be evaluated in this article, or claim that may be made by its manufacturer, is not guaranteed or endorsed by the publisher.
