# Supplementary material for: Apathy, Executive Function, and Emotion Recognition Are the Main Drivers of Functional Impairment in Behavioral Variant of Frontotemporal Dementia
Source: Front Neurol. 2022 Jan 13;12:734251. doi: 10.3389/fneur.2021.734251 (PMC8792989; doi:10.3389/fneur.2021.734251)
Supplement: Supplementary file 1 [file Data_Sheet_1.docx]

**Appendix**

**I. The Technology - Activities of Daily Living Questionnaire (T-ADLQ)**

*Instructions: Circle or cross one number for each item that better describes the ability of the patient for each activity described below.*

**1. Self-care activities**

*A. Eating*

0 = No problem

1 = Independent, but slow or some spills

2 = Needs help to cut or pour; spills often

3 = Must be fed most foods

9 = Don’t know

*B. Dressing*

0 = No problem

1 = Independent, but slow or clumsy

2 = Wrong sequence, forgets items

3 = Needs help with dressing

9 = Don’t know

*C. Bathing*

0 = No problem

1 = Bathes self, but needs to be reminded

2 = Bathes self with assistance

3 = Must be bathed by others

9 = Don’t know

*D. Elimination*

0 = Goes to the bathroom independently

1 = Goes to the bathroom when reminded; some accidents

2 = Needs assistance for elimination

3 = Has no control over either bowel or bladder

9 = Don’t know

*E. Taking pills or medicine*

0 = Remembers without help

1 = Remembers if dose is kept in a special place

2 = Needs spoken or written reminders

3 = Must be given medicine by others

9 = Does not take regular pills or medicine OR Don’t know

*F. Interest in personal appearance*

0 = Same as always

1 = Interested if going out, but not at home

2 = Allows self to be groomed, or does so on request only

3 = Resists efforts of caretaker to clean and groom

9 = Don’t know

**2. Household care**

*A. Preparing meals, cooking*

0 = Plans and prepares meals without difficulty

1 = Some cooking, but less than usual, or less variety

2 = Gets food only if it has already been prepared

3 = Does nothing to prepare meals

9 = Never did this activity OR Don’t know

*B. Setting the table*

0 = No problem

1 = Independent, but slow or clumsy

2 = Forgets items or puts them in the wrong place

3 = No longer does this activity

9 = Never did this activity OR Don’t know

*C. Housekeeping*

0 = Keeps house as usual

1 = Does at least half of his/her job

2 = Occasional dusting or small jobs

3 = No longer keeps house

9 = Never did this activity OR Don’t know

*D. Home maintenance*

0 = Does all of his/her usual tasks

1 = Does at least half of usual tasks

2 = Occasionally rakes or some other minor job

3 = No longer does any maintenance

9 = Never did this activity OR Don’t know

*E. Home repairs*

0 = Does all the usual repairs

1 = Does at least half of usual repairs

2 = Occasionally does minor repairs

3 = No longer does any repairs

9 = Never did this activity OR Don’t know

*F. Laundry*

0 = Does laundry as usual (same schedule, routine)

1 = Does laundry less frequently

2 = Does laundry only if reminded; leaves out detergent, steps

3 = No longer does laundry

9 = Never did this activity OR Don’t know

**3. Employment and recreation**

*A. Employment*

0 = Continues to work as usual

1 = Some mild problems with routine responsibilities

2 = Works at an easier job or part-time; threatened with loss of job

3 = No longer works

9 = Never worked OR retired before illness OR Don’t know

*B. Recreation*

0 = Same as usual

1 = Engages in recreational activities less frequently

2 = Has lost some skills necessary for recreational activities (eg, bridge, golfing); needs coaxing to participate

3 = No longer pursues recreational activities

9 = Never engaged in recreational activities OR Don’t know

*C. Organizations*

0 = Attends meetings, takes responsibilities as usual

1 = Attends less frequently

2 = Attends occasionally; has no major responsibilities

3 = No longer attends

9 = Never participated in organizations OR Don’t know

*D. Travel*

0 = Same as usual

1 = Gets out if someone else drives

2 = Gets out in wheelchair

3 = Home- or hospital-bound

9 = Don’t know

**4. Shopping and money**

*A. Food shopping*

0 = No problem

1 = Forgets items or buys unnecessary items

2 = Needs to be accompanied while shopping

3 = No longer does the shopping

9 = Never had responsibility in this activity OR Don’t know

*B. Handling cash*

0 = No problem

1 = Has difficulty paying proper amount, counting

2 = Loses or misplaces money

3 = No longer handles money

9 = Never had responsibility for this activity OR Don’t know

*C. Managing finances*

0 = No problem paying bills, banking

1 = Pays bills late; some trouble writing checks

2 = Forgets to pay bills; has trouble balancing checkbook; needs help from others

3 = No longer manages finances

9 = Never had responsibility in this activity OR Don’t know

**5. Travel**

*A. Public transportation*

0 = Uses public transportation as usual

1 = Uses public transportation less frequently

2 = Has gotten lost using public transportation

3 = No longer uses public transportation

9 = Never used public transportation regularly OR Don’t know

*B. Driving*

0 = Drives as usual

1 = Drives more cautiously

2 = Drives less carefully; has gotten lost while driving

3 = No longer drives

9 = Never drove OR Don’t know

*C. Mobility around the neighborhood*

0 = Same as usual

1 = Goes out less frequently

2 = Has gotten lost in the immediate neighborhood

3 = No longer goes out unaccompanied

9 = This activity has been restricted in the past OR Don’t know

*D. Travel outside familiar environment*

0 = Same as usual

1 = Occasionally gets disoriented in strange surroundings

2 = Gets very disoriented but is able to manage if accompanied

3 = No longer able to travel

9 = Never did this activity OR Don’t know

**6. Communication**

*A. Using the telephone*

0 = Same as usual

1 = Calls a few familiar numbers

2 = Will only answer telephone (won’t make calls)

3 = Does not use the telephone at all

9 = Never had a telephone OR Don’t know

*B. Talking*

0 = Same as usual

1 = Less talkative; has trouble thinking of words or names

2 = Makes occasional errors in speech

3 = Speech is almost unintelligible

9 = Don’t know

*C. Understanding*

0 = Understands everything that is said as usual

1 = Asks for repetition

2 = Has trouble understanding conversations or specific words occasionally

3 = Does not understand what people are saying most of the time

9 = Don’t know

*D. Reading*

0 = Same as usual

1 = Reads less frequently

2 = Has trouble understanding or remembering what he/she has read

3 = Has given up reading

9 = Never read much OR Don’t know

*E. Writing*

0 = Same as usual

1 = Writes less often; makes occasional spelling errors

2 = Signs name but no other writing

3 = Never writes

9 = Never wrote much OR Don’t know

**6. Technology**

*A. Computer use*

0 = Same as usual

1 = Can turn on the computer and perform basic tasks

2 = Only remembers how to turn the computer on or off

3 = Can’t use the computer anymore

9 = Never used the computer OR Don’t know

*B. Cell phone use*

0 = Same us usual

1 = Able to answer or make calls on the cellphone

2 = Has difficulties answering calls

3 = Doesn’t know how to use it anymore

9 = Never used a cellphone OR Don’t know

*C. ATM use*

0 = Same as usual

1 = Has some troubles withdrawing money from the ATM

2 = Doesn’t remember his/her own password

3 = Can’t use the ATM anymore

9 = Never used the ATM OR Don’t know

*D. Internet access*

0 = Same as usual

1 = Needs help navigating the internet

2 = Forget passwords for several websites

3 = Doesn’t use internet anymore

9 = Never used OR Don’t know

*E. Mail use*

0 = Same as usual

1 = Able to answer and check the mail, but has difficulties performing advanced tasks (e.g. Attach Data)

2 = Doesn’t remember mail password or website

3 = Not able to use the mail to communicate

9 = Never used mail OR Don’t know

*F. Social network*

0 = Same as usual

1 = Able to perform basic social network tasks, but has difficulties performing advanced tasks (e.g. Upload a picture or publish a comment)

2 = Able to use social networks only with help

3 = No longer able to use social networks

9 = Never used social networks OR Don’t know

**Score Equation of The Technology - Activities of Daily Living Questionnaire (T-ADLQ)**

⅀ Total Score

*[Except items with 9 as score]*

x100

(3 x numbers of items answered)

*[Except items with 9 as score]*


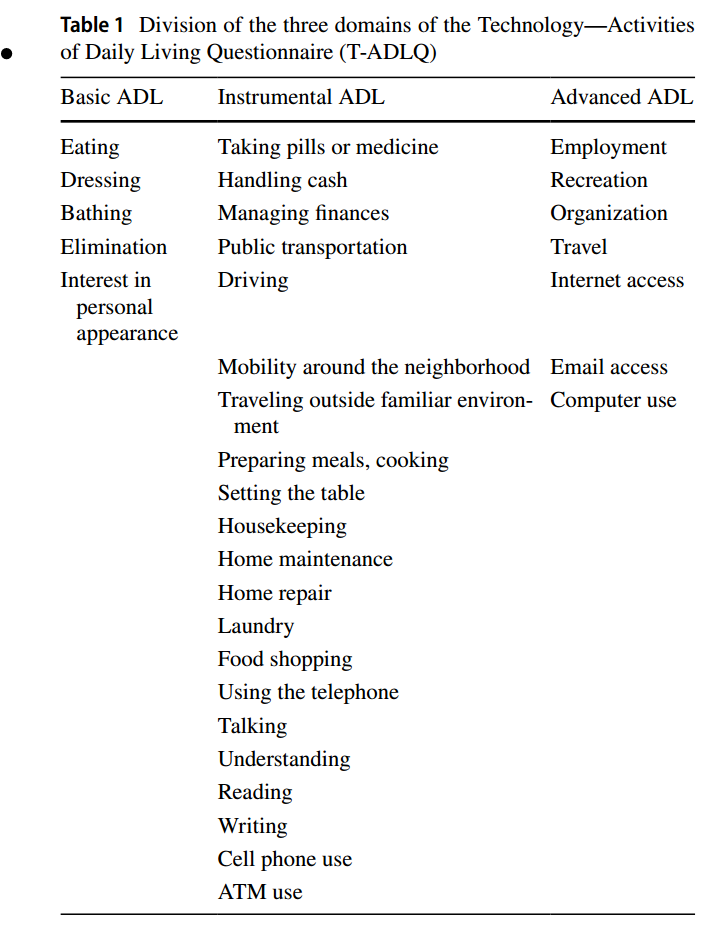


Table from Slachevsky et al., (2019).

**II. Composite scores for the mini-Social cognition & Emotional Assessment (mini-SEA)**

1. **Faux pas**

Stories with a faux pas: ___/30 points

Stories without a faux pas: ___/10 points

Total Faux pas score: ___/40 points

Now, transform the raw score into a composite score with the following formula:

⅀ Total Faux pas Score

x 1,5

4

Total Faux pas composite score: ___/15 points

1. **Emotion recognition**

Transform the raw score (number of emotions correctly recognized) into a composite score with the following formula:

⅀ Total Faux pas Score

x 1,5

3,5

Total Emotion recognition composite score: ___/15 points

1. **Total score mini-SEA**

Faux pas composite score + Emotion recognition composite score

Total mini-SEA score: ___/30 points
