# Supplementary material for: Apathy, Executive Function, and Emotion Recognition Are the Main Drivers of Functional Impairment in Behavioral Variant of Frontotemporal Dementia
Source: Front Neurol. 2022 Jan 13;12:734251. doi: 10.3389/fneur.2021.734251 (PMC8792989; doi:10.3389/fneur.2021.734251)
Supplement: Supplementary file 2 [file Table_1.DOCX]

#### Table 1

#### *Standard multiple regression analyses with the percentage of functional impairment for basic activities of daily living (BADLs), instrumental activities of daily living (IADLs) and advanced activities of daily living (a-ADLs) scores as dependent variables*

| *Predictor* | *B* | 95% *CI* for *B* | | *SE* for *B* | *β* | *p-*value | *sr^2^* |
| --- | --- | --- | --- | --- | --- | --- | --- |
|  |  | *LL* | *UL* |  |  |  |  |
| Basic ADLs (BADLs) |  |  |  |  |  |  |  |
| Constant | 0.79 | -4.68 | 6.26 | 2.73 |  |  |  |
| Apathy | 11.82 | 1.65 | 25.55 | 5.06 | 0.33 | .02 | 0.09 |
| Disinhibition | 11.35 | 0.47 | 21.79 | 5.42 | 0.29 | .04 | 0.08 |
| Instrumental ADLs (IADLs) |  |  |  |  |  |  |  |
| Constant | -18.79 | -63.09 | 25.50 | 22.05 |  |  |  |
| Executive Function | -1.59 | -3.59 | -0.50 | 0.75 | -0.30 | .04 | 0.04 |
| Social Cognition | 1.28 | -0.69 | 2.76 | 0.86 | 0.21 | .14 | 0.04 |
| Apathy | 38.75 | 23.43 | 48.56 | 6.48 | 0.70 | < .001 | 0.42 |
| Advanced ADLs (a-ADLs) |  |  |  |  |  |  |  |
| Constant | 9.24 | -39.60 | 58.01 | 24.32 |  |  |  |
| Executive Function | -1.31 | -2.91 | 0.50 | 0.82 | -0.24 | .11 | 0.05 |
| Social Cognition | 1.14 | -1.68 | 2.13 | 0.94 | 0.18 | .23 | 0.03 |
| Apathy | 38.89 | 19.27 | 46.97 | 7.05 | 0.69 | < .001 | 0.38 |

#### *B* = Unstandardized regression coefficient; CI = Confidence interval; *LL* = Lower limit; *UL* = Upper limit; *SE* = Standard error; *β* = Standardized coefficient; s*r^2^*= Semi-partial correlation squared

#### *Note:* adjusted *R^2^* for BADLs = .282, *p*< .001; adjusted *R^2^* for IADLs = .588, *p*< .001; adjusted *R^2^* for a-ADLs = .529, *p*< .001
